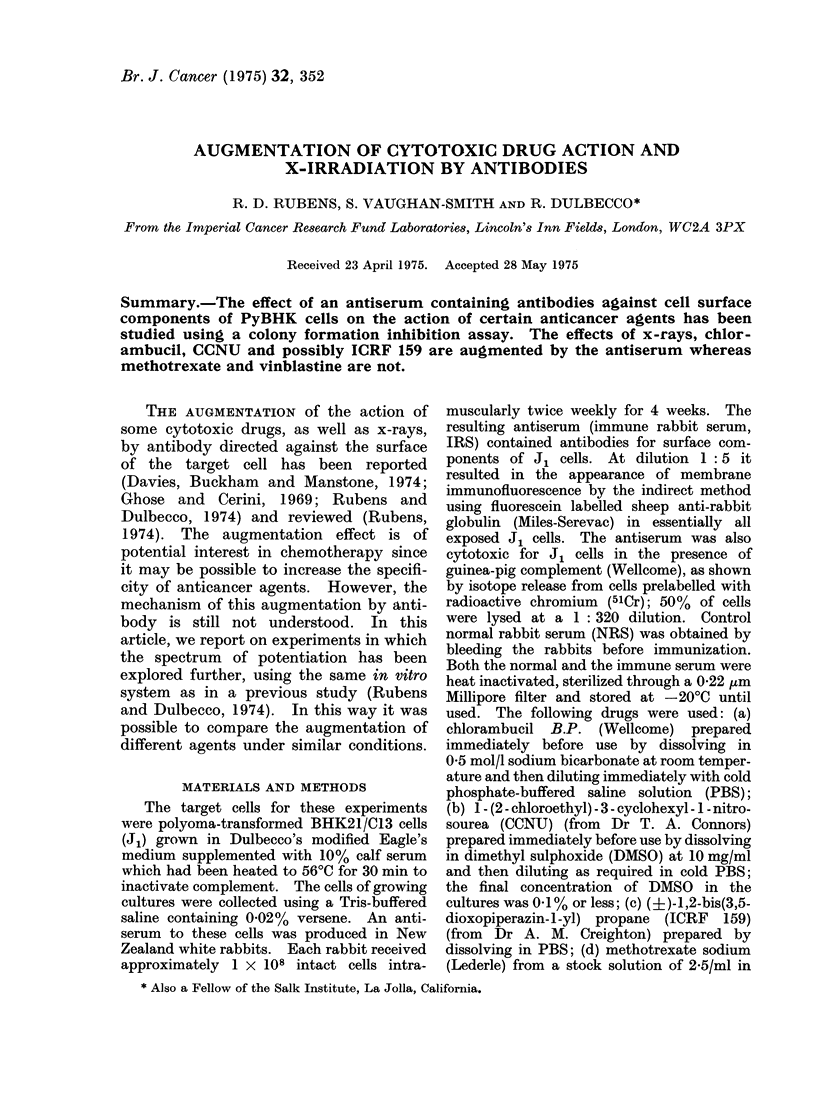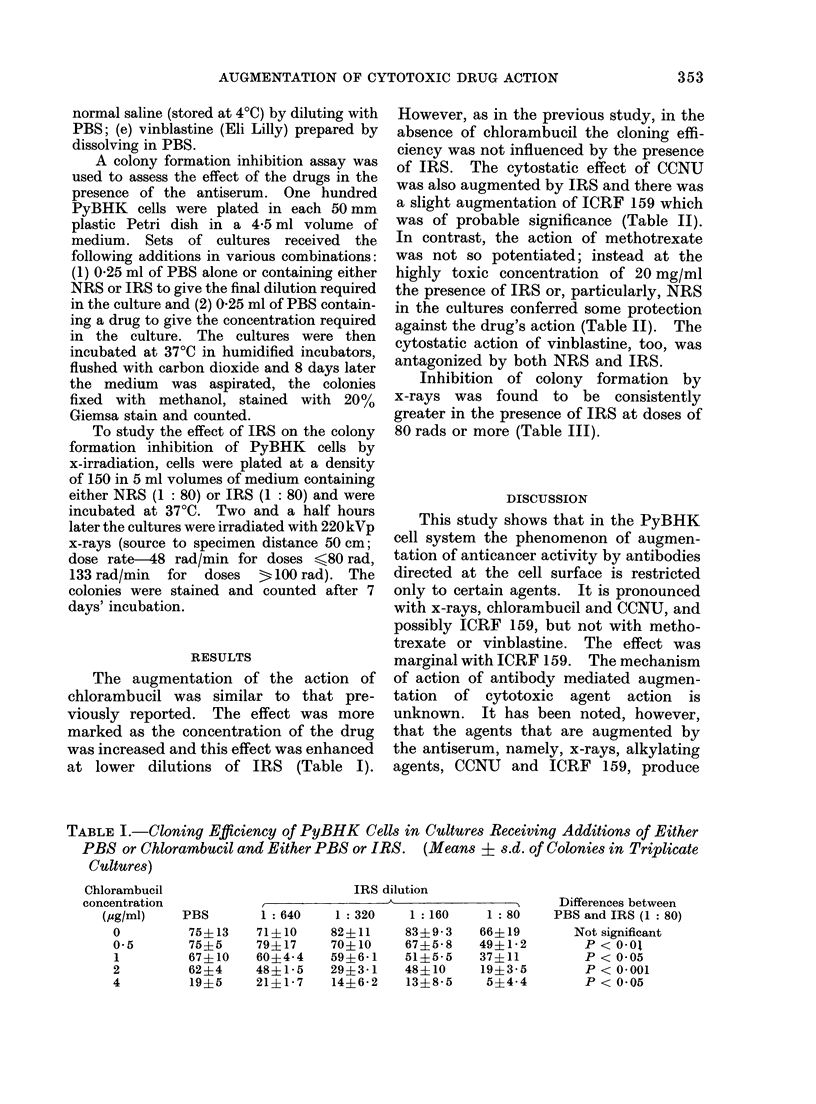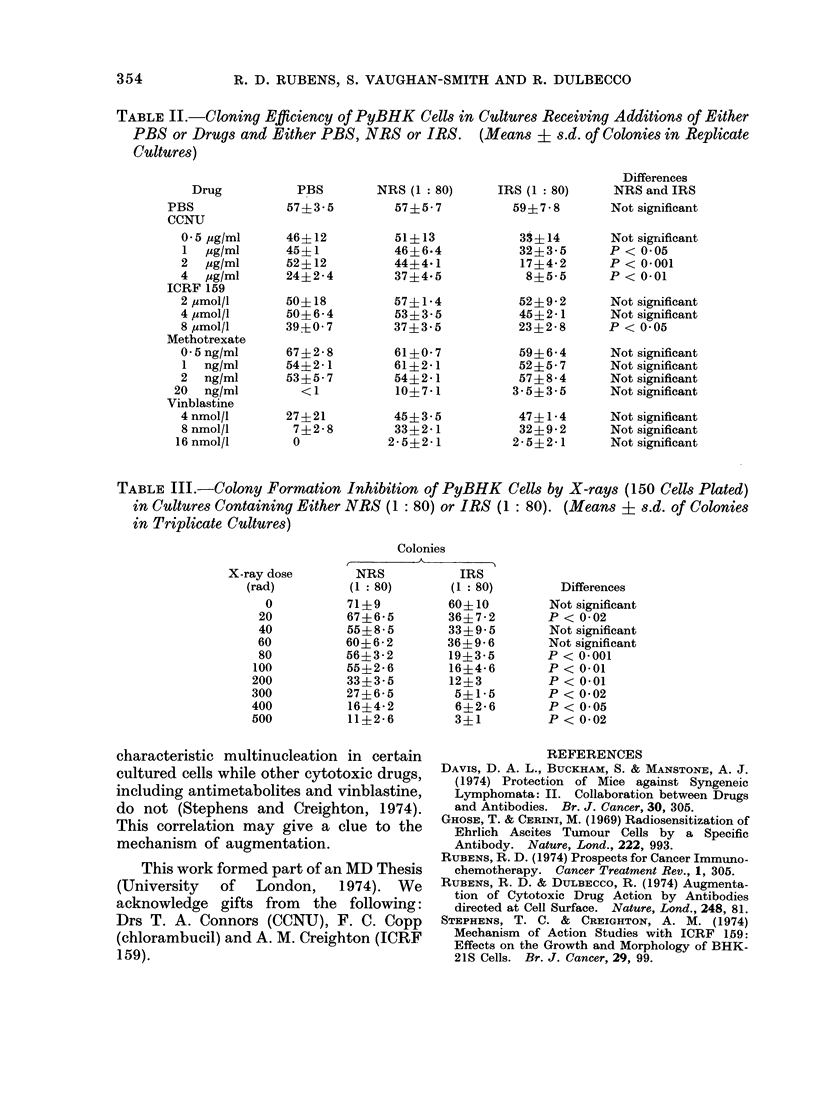# Augmentation of cytotoxic drug action and X-irradiation by antibodies.

**DOI:** 10.1038/bjc.1975.234

**Published:** 1975-09

**Authors:** R. D. Rubens, S. Vaughan-Smith, R. Dulbecco

## Abstract

The effect of an antiserum containing antibodies against cell surface components of PyBHK cells on the action of certain anticancer agents has been studied using a colony formation inhibition assay. The effects of x-rays, chlorambucil, CCNU and possibly ICRF 159 are augmented by the antiserum whereas methotrexate and vinblastine are not.


*Br. J. Cancer* (1975) **32**, 352

### AUGMENTATION OF CYTOTOXIC DRUG ACTION AND X-IRRADIATION BY ANTIBODIES

R. D. RUBENS, S. VAUGHAN-SMITH AND R. DULBECCO*

*From the Imperial Cancer Research Fund Laboratories, Lincoln's Inn Fields, London, WC2A 3PX*

Received 23 April 1975. Accepted 28 May 1975

**Summary.—The effect of an antiserum containing antibodies against cell surface components of PyBHK cells on the action of certain anticancer agents has been studied using a colony formation inhibition assay. The effects of x-rays, chlorambucil, CCNU and possibly ICRF 159 are augmented by the antiserum whereas methotrexate and vinblastine are not.**

THE AUGMENTATION of the action of some cytotoxic drugs, as well as x-rays, by antibody directed against the surface of the target cell has been reported (Davies, Buckham and Manstone, 1974; Ghose and Cerini, 1969; Rubens and Dulbecco, 1974) and reviewed (Rubens, 1974). The augmentation effect is of potential interest in chemotherapy since it may be possible to increase the specificity of anticancer agents. However, the mechanism of this augmentation by antibody is still not understood. In this article, we report on experiments in which the spectrum of potentiation has been explored further, using the same *in vitro* system as in a previous study (Rubens and Dulbecco, 1974). In this way it was possible to compare the augmentation of different agents under similar conditions.

### MATERIALS AND METHODS

The target cells for these experiments were polyoma-transformed BHK21/C13 cells ($J_1$) grown in Dulbecco's modified Eagle's medium supplemented with 10% calf serum which had been heated to 56°C for 30 min to inactivate complement. The cells of growing cultures were collected using a Tris-buffered saline containing 0·02% versene. An antiserum to these cells was produced in New Zealand white rabbits. Each rabbit received approximately $1 \times 10^8$ intact cells intramuscularly twice weekly for 4 weeks. The resulting antiserum (immune rabbit serum, IRS) contained antibodies for surface components of $J_1$ cells. At dilution 1 : 5 it resulted in the appearance of membrane immunofluorescence by the indirect method using fluorescein labelled sheep anti-rabbit globulin (Miles-Serevac) in essentially all exposed $J_1$ cells. The antiserum was also cytotoxic for $J_1$ cells in the presence of guinea-pig complement (Wellcome), as shown by isotope release from cells prelabelled with radioactive chromium ($^{51}Cr$); 50% of cells were lysed at a 1 : 320 dilution. Control normal rabbit serum (NRS) was obtained by bleeding the rabbits before immunization. Both the normal and the immune serum were heat inactivated, sterilized through a 0·22 $\mu$m Millipore filter and stored at −20°C until used. The following drugs were used: (a) chlorambucil *B.P.* (Wellcome) prepared immediately before use by dissolving in 0·5 mol/l sodium bicarbonate at room temperature and then diluting immediately with cold phosphate-buffered saline solution (PBS); (b) 1-(2-chloroethyl)-3-cyclohexyl-1-nitrosourea (CCNU) (from Dr T. A. Connors) prepared immediately before use by dissolving in dimethyl sulphoxide (DMSO) at 10 mg/ml and then diluting as required in cold PBS; the final concentration of DMSO in the cultures was 0·1% or less; (c) (±)-1,2-bis(3,5-dioxopiperazin-1-yl) propane (ICRF 159) (from Dr A. M. Creighton) prepared by dissolving in PBS; (d) methotrexate sodium (Lederle) from a stock solution of 2·5/ml in

* Also a Fellow of the Salk Institute, La Jolla, California.

normal saline (stored at 4°C) by diluting with PBS; (e) vinblastine (Eli Lilly) prepared by dissolving in PBS.

A colony formation inhibition assay was used to assess the effect of the drugs in the presence of the antiserum. One hundred PyBHK cells were plated in each 50 mm plastic Petri dish in a 4·5 ml volume of medium. Sets of cultures received the following additions in various combinations: (1) 0·25 ml of PBS alone or containing either NRS or IRS to give the final dilution required in the culture and (2) 0·25 ml of PBS containing a drug to give the concentration required in the culture. The cultures were then incubated at 37°C in humidified incubators, flushed with carbon dioxide and 8 days later the medium was aspirated, the colonies fixed with methanol, stained with 20% Giemsa stain and counted.

To study the effect of IRS on the colony formation inhibition of PyBHK cells by x-irradiation, cells were plated at a density of 150 in 5 ml volumes of medium containing either NRS (1 : 80) or IRS (1 : 80) and were incubated at 37°C. Two and a half hours later the cultures were irradiated with 220 kVp x-rays (source to specimen distance 50 cm; dose rate—48 rad/min for doses $\leqslant$80 rad, 133 rad/min for doses $\geqslant$100 rad). The colonies were stained and counted after 7 days' incubation.

### RESULTS

The augmentation of the action of chlorambucil was similar to that previously reported. The effect was more marked as the concentration of the drug was increased and this effect was enhanced at lower dilutions of IRS (Table I). However, as in the previous study, in the absence of chlorambucil the cloning efficiency was not influenced by the presence of IRS. The cytostatic effect of CCNU was also augmented by IRS and there was a slight augmentation of ICRF 159 which was of probable significance (Table II). In contrast, the action of methotrexate was not so potentiated; instead at the highly toxic concentration of 20 mg/ml the presence of IRS or, particularly, NRS in the cultures conferred some protection against the drug's action (Table II). The cytostatic action of vinblastine, too, was antagonized by both NRS and IRS.

Inhibition of colony formation by x-rays was found to be consistently greater in the presence of IRS at doses of 80 rads or more (Table III).

### DISCUSSION

This study shows that in the PyBHK cell system the phenomenon of augmentation of anticancer activity by antibodies directed at the cell surface is restricted only to certain agents. It is pronounced with x-rays, chlorambucil and CCNU, and possibly ICRF 159, but not with methotrexate or vinblastine. The effect was marginal with ICRF 159. The mechanism of action of antibody mediated augmentation of cytotoxic agent action is unknown. It has been noted, however, that the agents that are augmented by the antiserum, namely, x-rays, alkylating agents, CCNU and ICRF 159, produce

TABLE I.—*Cloning Efficiency of PyBHK Cells in Cultures Receiving Additions of Either PBS or Chlorambucil and Either PBS or IRS. (Means ± s.d. of Colonies in Triplicate Cultures)*

| Chlorambucil concentration (μg/ml) | PBS | IRS dilution 1 : 640 | 1 : 320 | 1 : 160 | 1 : 80 | Differences between PBS and IRS (1 : 80) |
|---|---|---|---|---|---|---|
| 0 | 75±13 | 71±10 | 82±11 | 83±9·3 | 66±19 | Not significant |
| 0·5 | 75±5 | 79±17 | 70±10 | 67±5·8 | 49±1·2 | $P < 0·01$ |
| 1 | 67±10 | 60±4·4 | 59±6·1 | 51±5·5 | 37±11 | $P < 0·05$ |
| 2 | 62±4 | 48±1·5 | 29±3·1 | 48±10 | 19±3·5 | $P < 0·001$ |
| 4 | 19±5 | 21±1·7 | 14±6·2 | 13±8·5 | 5±4·4 | $P < 0·05$ |

TABLE II.—*Cloning Efficiency of PyBHK Cells in Cultures Receiving Additions of Either PBS or Drugs and Either PBS, NRS or IRS. (Means ± s.d. of Colonies in Replicate Cultures)*

| Drug | PBS | NRS (1 : 80) | IRS (1 : 80) | Differences NRS and IRS |
|---|---|---|---|---|
| PBS | 57±3·5 | 57±5·7 | 59±7·8 | Not significant |
| CCNU | | | | |
| 0·5 μg/ml | 46±12 | 51±13 | 33±14 | Not significant |
| 1 μg/ml | 45±1 | 46±6·4 | 32±3·5 | $P < 0·05$ |
| 2 μg/ml | 52±12 | 44±4·1 | 17±4·2 | $P < 0·001$ |
| 4 μg/ml | 24±2·4 | 37±4·5 | 8±5·5 | $P < 0·01$ |
| ICRF 159 | | | | |
| 2 μmol/l | 50±18 | 57±1·4 | 52±9·2 | Not significant |
| 4 μmol/l | 50±6·4 | 53±3·5 | 45±2·1 | Not significant |
| 8 μmol/l | 39±0·7 | 37±3·5 | 23±2·8 | $P < 0·05$ |
| Methotrexate | | | | |
| 0·5 ng/ml | 67±2·8 | 61±0·7 | 59±6·4 | Not significant |
| 1 ng/ml | 54±2·1 | 61±2·1 | 52±5·7 | Not significant |
| 2 ng/ml | 53±5·7 | 54±2·1 | 57±8·4 | Not significant |
| 20 ng/ml | <1 | 10±7·1 | 3·5±3·5 | Not significant |
| Vinblastine | | | | |
| 4 nmol/l | 27±21 | 45±3·5 | 47±1·4 | Not significant |
| 8 nmol/l | 7±2·8 | 33±2·1 | 32±9·2 | Not significant |
| 16 nmol/l | 0 | 2·5±2·1 | 2·5±2·1 | Not significant |

TABLE III.—*Colony Formation Inhibition of PyBHK Cells by X-rays* (150 *Cells Plated*) *in Cultures Containing Either NRS* (1 : 80) *or IRS* (1 : 80). (*Means ± s.d. of Colonies in Triplicate Cultures*)

| X-ray dose (rad) | Colonies NRS (1 : 80) | IRS (1 : 80) | Differences |
|---|---|---|---|
| 0 | 71±9 | 60±10 | Not significant |
| 20 | 67±6·5 | 36±7·2 | $P < 0·02$ |
| 40 | 55±8·5 | 33±9·5 | Not significant |
| 60 | 60±6·2 | 36±9·6 | Not significant |
| 80 | 56±3·2 | 19±3·5 | $P < 0·001$ |
| 100 | 55±2·6 | 16±4·6 | $P < 0·01$ |
| 200 | 33±3·5 | 12±3 | $P < 0·01$ |
| 300 | 27±6·5 | 5±1·5 | $P < 0·02$ |
| 400 | 16±4·2 | 6±2·6 | $P < 0·05$ |
| 500 | 11±2·6 | 3±1 | $P < 0·02$ |

characteristic multinucleation in certain cultured cells while other cytotoxic drugs, including antimetabolites and vinblastine, do not (Stephens and Creighton, 1974). This correlation may give a clue to the mechanism of augmentation.

This work formed part of an MD Thesis (University of London, 1974). We acknowledge gifts from the following: Drs T. A. Connors (CCNU), F. C. Copp (chlorambucil) and A. M. Creighton (ICRF 159).

### REFERENCES

DAVIS, D. A. L., BUCKHAM, S. & MANSTONE, A. J. (1974) Protection of Mice against Syngeneic Lymphomata: II. Collaboration between Drugs and Antibodies. *Br. J. Cancer*, **30**, 305.

GHOSE, T. & CERINI, M. (1969) Radiosensitization of Ehrlich Ascites Tumour Cells by a Specific Antibody. *Nature, Lond.*, **222**, 993.

RUBENS, R. D. (1974) Prospects for Cancer Immunochemotherapy. *Cancer Treatment Rev.*, **1**, 305.

RUBENS, R. D. & DULBECCO, R. (1974) Augmentation of Cytotoxic Drug Action by Antibodies directed at Cell Surface. *Nature, Lond.*, **248**, 81.

STEPHENS, T. C. & CREIGHTON, A. M. (1974) Mechanism of Action Studies with ICRF 159: Effects on the Growth and Morphology of BHK-21S Cells. *Br. J. Cancer*, **29**, 99.